# Occult Esophageal Squamous Cell Carcinoma Presenting as Bone-Predominant Carcinoma of Unknown Primary: A Case Report of a p40-Negative Vertebral Metastasis

**DOI:** 10.3390/diagnostics16162677

**Published:** 2026-08-21

**Authors:** Hassan Brim, Wardah Bajwa, Anas Brim, Farshad Aduli, Amro AbdelLatief, Rabia Zafar, Adeyinka O. Laiyemo, Hassan Ashktorab

**Affiliations:** 1 Department of Medicine, GI Division, Cancer Center, Howard University Hospital, Washington, DC 20060, USA; hbrim@howard.edu (H.B.); drwardabajwa@gmail.com (W.B.); anas.brim1@howard.edu (A.B.); farshad.aduli@howard.edu (F.A.); aabdellatief@huhosp.org (A.A.); rabia.zafar@howard.edu (R.Z.); adeyinka.laiyemo@howard.edu (A.O.L.); 2 Department of Pathology, Howard University, Washington, DC 20060, USA

**Keywords:** esophageal squamous cell carcinoma, immunohistochemistry, p40-negative staining, carcinoma of unknown primary, bone metastasis, bone biopsy, diagnostic pitfalls

## Abstract

**Background and Clincal significance:** Esophageal squamous cell carcinoma (ESCC) classically presents with progressive dysphagia and weight loss, but atypical presentations may redirect the diagnostic workup before the esophageal primary is identified. We report a case illustrating the simultaneous convergence of three diagnostic pitfalls: absence of dysphagia, bone-predominant metastatic presentation initially managed as carcinoma of unknown primary (CUP), and negative p40 staining in a vertebral biopsy in a patient subsequently confirmed to have invasive mid-esophageal squamous cell carcinoma. **Case** **Presentation:** A 66-year-old African American man with dementia, active tobacco exposure, and prior alcohol use disorder presented with constipation, abdominal pain, melena, fever, nausea, vomiting, and progressive back pain. Dysphagia or odynophagia was not documented. CT of the abdomen and pelvis demonstrated diffuse lytic osseous metastases. During evaluation and palliation of symptomatic L2 disease, kyphoplasty and radiofrequency ablation were performed, and bilateral core biopsies showed poorly differentiated carcinoma that was AE1/AE3-positive but negative for p40, CK7, CK20, TTF-1, S100, GATA-3, PAX8, and NKX3.1, yielding an initial diagnosis of CUP. Subsequent chest CT revealed esophageal wall thickening with intraluminal debris. Esophagogastroduodenoscopy (EGD) identified a non-obstructive ulcerated mid-esophageal lesion, and biopsy confirmed invasive squamous cell carcinoma. Poor performance status precluded systemic therapy; palliative external-beam radiation was initiated after diagnosis but discontinued because of clinical deterioration, and the patient transitioned to hospice before passing several weeks after diagnosis. Melena is a gastrointestinal alarm feature, and the combination of gastrointestinal bleeding and an esophageal imaging abnormality warrants timely endoscopic evaluation even when dysphagia is not reported or the symptom history is unreliable. Negative p40 staining in a poorly differentiated, potentially decalcified bone specimen may reflect loss of lineage-marker expression, technical antigen degradation, or both. Tissue or plasma genomic profiling and emerging cell-free DNA methylation classifiers could complement the workup but would not replace direct biopsy of a radiographically suspicious esophageal lesion. **Conclusions:** In metastatic poorly differentiated carcinoma, lack of documented dysphagia should not exclude an esophageal primary, particularly in patients with cognitive impairment. A p40-negative bone biopsy does not rule out squamous lineage. Timely EGD and integrated clinicopathologic assessment are essential when clinical or imaging findings suggest esophageal involvement.

## 1. Introduction

Esophageal squamous cell carcinoma (ESCC) is an aggressive malignancy with a five-year overall survival below 20% at advanced stage [1]. Globally, ESCC accounts for the majority of esophageal cancers, though its relative frequency in the United States has declined with the rising incidence of esophageal adenocarcinoma (EAC) [2]. The two histotypes differ in anatomic distribution, risk factor profile, and demographics. ESCC most often arises in the middle or upper esophagus and is strongly associated with tobacco use and heavy alcohol consumption, whereas EAC predominates in the distal esophagus and GEJ in the setting of reflux disease and obesity [2,3].

In the United States, ESCC continues to impose a disproportionate burden on African American men, who experience higher incidence rates and worse survival than non-Hispanic White patients even after adjustment for stage and treatment [4,5,6]. Persistent disparities in access to timely endoscopic evaluation and treatment delivery amplify this gap [4,5].

Progressive dysphagia and weight loss are hallmark presenting symptoms of ESCC, reported in the vast majority of symptomatic patients [1,7]. However, these symptoms are not invariable, and the absence of reported dysphagia may be unreliable in patients with cognitive impairment. A small, non-obstructive, or ulcerated primary lesion may not produce mechanical symptoms, while melena or other gastrointestinal symptoms may provide an alternative clue [1]. When diffuse osseous metastases are the dominant finding, the differential diagnosis typically encompasses hematologic malignancies, metastatic prostate carcinoma, and CUP rather than a luminal gastrointestinal tumor [8,9]. Immunohistochemistry (IHC) is central to the workup of poorly differentiated metastatic carcinoma. p40, targeting the ΔNp63 isoform, is the most specific available marker of squamous differentiation, preferred over p63 for this purpose [10,11]. Nevertheless, its sensitivity is not absolute: poorly differentiated tumors may lose lineage-specific marker expression, and bone biopsies subjected to decalcification protocols are susceptible to antigen degradation that can produce false-negative results [12,13]. Failure to recognize this limitation may redirect the workup away from an occult squamous primary.

We report a 66-year-old African American man with stage IV mid-esophageal ESCC whose presentation was dominated by diffuse lytic bone metastases. Although dysphagia was not documented, dementia limited confidence in the symptom history, and the patient had melena and abdominal symptoms. An initial vertebral biopsy was AE1/AE3-positive but p40-negative, leading to a working diagnosis of CUP. Definitive diagnosis required EGD after chest CT demonstrated esophageal wall thickening. This case highlights the convergence of a bone-predominant metastatic presentation, a p40-negative metastatic biopsy, and incomplete symptom characterization, emphasizing that gastrointestinal alarm features and site-specific imaging abnormalities should prompt direct endoscopic assessment.

## 2. Case Presentation

A 66-year-old African American man with hypertension, asthma, and traumatic brain injury complicated by dementia and chronic gait disturbance presented to the emergency department with four to five days of constipation, diffuse abdominal pain, nausea, vomiting, melena, and low-grade fever. He had a 30 pack-year smoking history and was an active smoker at diagnosis. He also had prior alcohol use disorder, reportedly in long-term remission, and used marijuana one to two times weekly. Dysphagia or odynophagia was not documented in the available history; however, because of dementia, the reliability of negative symptom reporting was uncertain. Initial laboratory evaluation was unrevealing; no imaging or endoscopy was performed, and he was discharged after symptomatic improvement with laxatives. The available record did not document the rationale for deferring endoscopic evaluation at this visit.

Two weeks later, he presented to a second emergency department with worsening constipation, diffuse abdominal pain, and new lumbar and sacral back pain. CT of the abdomen and pelvis demonstrated innumerable lytic osseous lesions involving the axial and appendicular skeleton, highly suspicious for metastatic malignancy, as well as prostatic enlargement. He was transferred to a tertiary center for further evaluation. At this stage, chest imaging had not yet identified an esophageal abnormality, and the dominant clinical problem was symptomatic diffuse skeletal disease.

**Figure 1:**
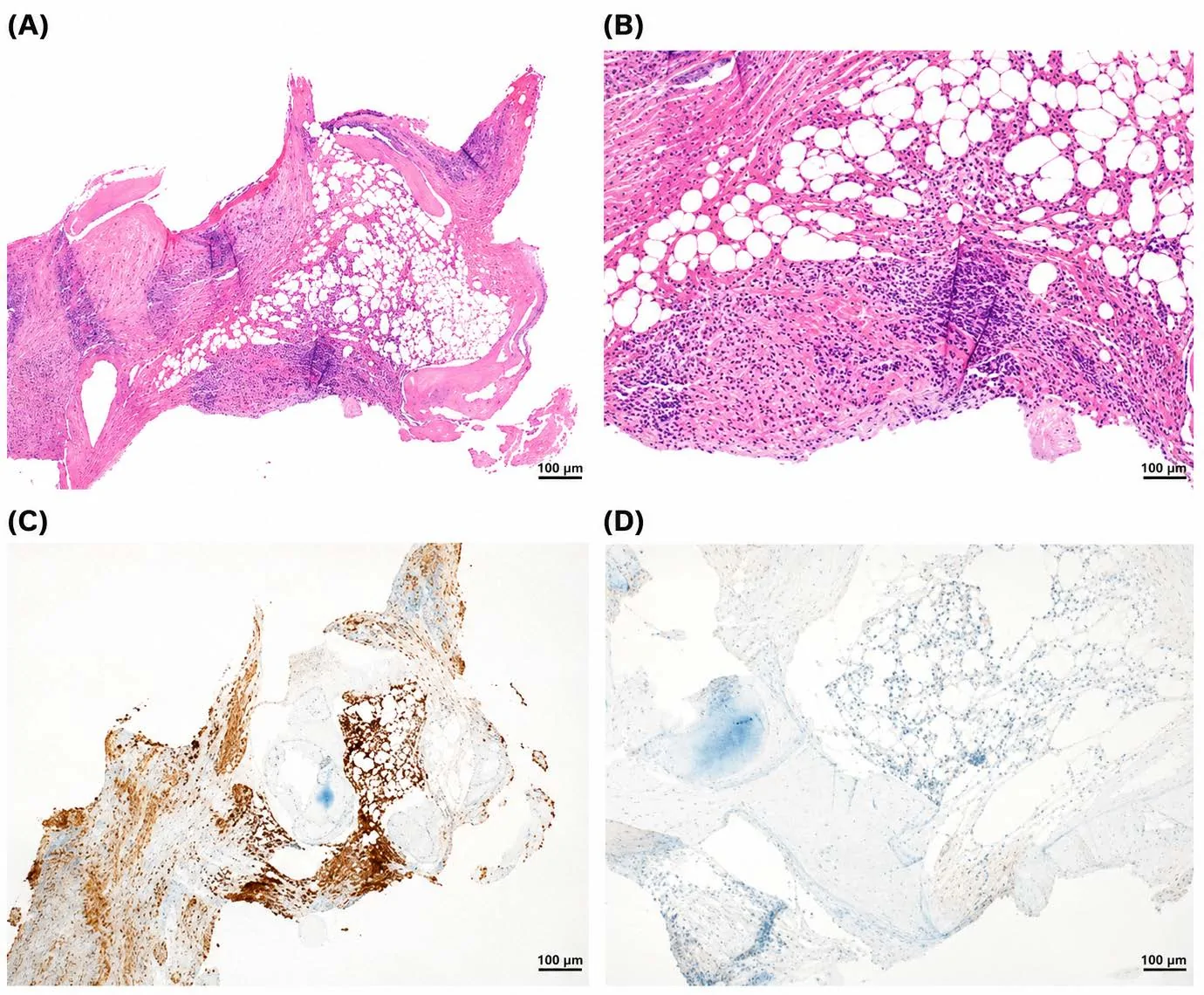
Histopathology and immunohistochemistry of the L2 vertebral metastasis. (**A**) Low-power (40×) H&E staining demonstrates infiltration of bone marrow by poorly differentiated carcinoma. (**B**) Higher magnification (100×) highlights malignant cellular morphology. (**C**) Diffuse AE1/AE3 positivity confirms epithelial origin. (**D**) Complete absence of p40 nuclear staining in the vertebral bone biopsy specimen.

At the tertiary center, the symptomatic L2 lesion was selected for local intervention and tissue sampling. He underwent L2 kyphoplasty and radiofrequency ablation, with bilateral core biopsies obtained during the same procedural episode. Radiofrequency ablation was a local interventional procedure and was distinct from the external-beam radiation therapy administered later after the ESCC diagnosis. Histopathology showed poorly differentiated carcinoma infiltrating bone marrow. An extensive IHC panel demonstrated diffuse AE1/AE3 positivity, confirming epithelial origin, with negative staining for CK7, CK20, thyroid transcription factor-1 (TTF-1), p40, S100, GATA-3, PAX8, and NKX3.1. No lineage-specific differentiation could be established, and the diagnosis was designated metastatic CUP. Representative histopathologic and IHC findings are shown in Figure 1.

**Figure 2:**
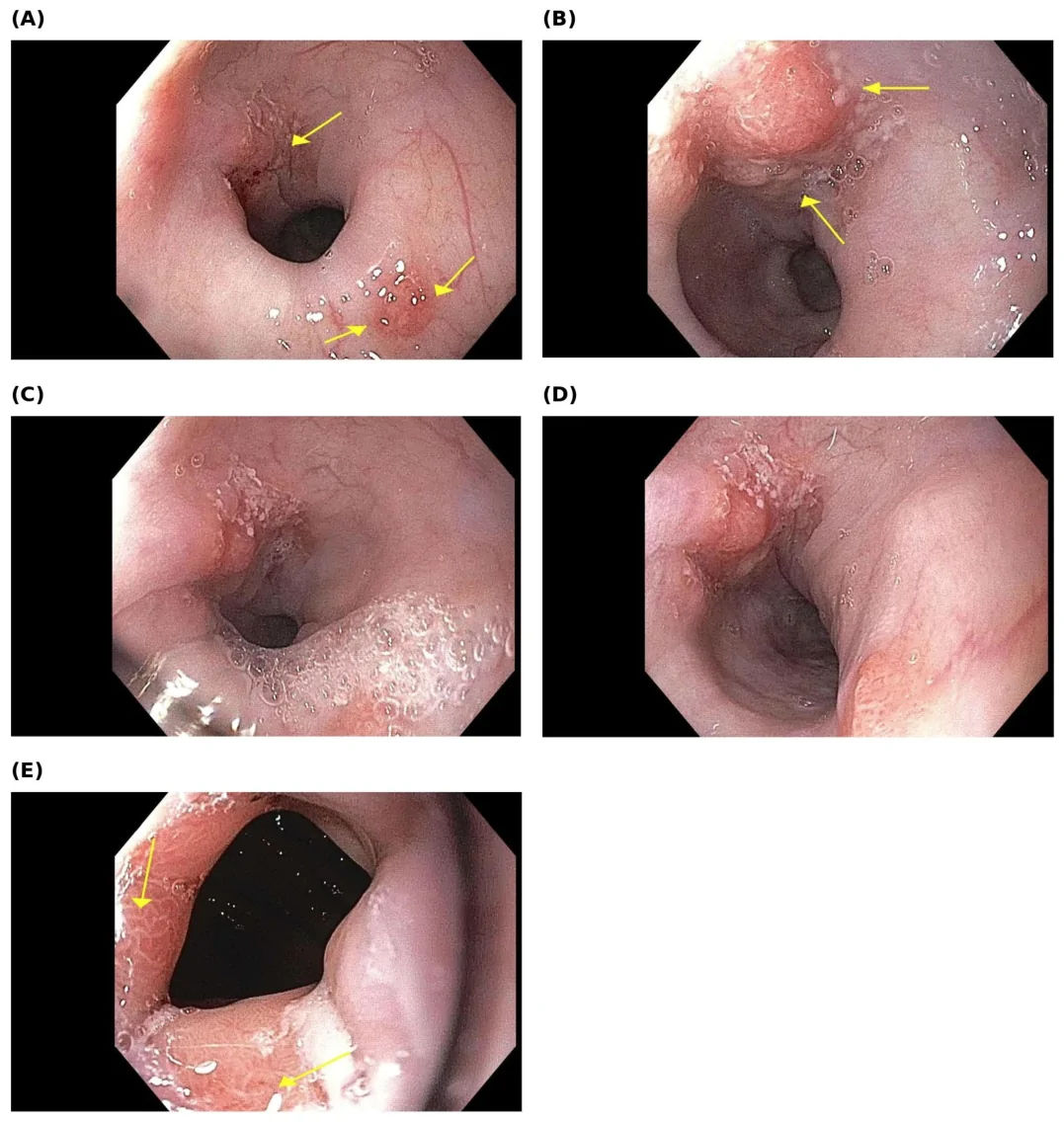
Endoscopic findings at esophagogastroduodenoscopy. (**A**) Ulcerated, erythematous mucosal abnormality (arrow) in the middle third of the esophagus, including a small clean-based ulcer, without luminal obstruction (**B**–**D**). (**E**) Inflammatory changes consistent with reflux esophagitis at the gastroesophageal junction.

Subsequent CT of the chest confirmed diffuse osseous metastatic disease and demonstrated segmental esophageal wall thickening with intraluminal debris, providing the first site-specific imaging clue to an esophageal primary. Oncologic evaluation also identified small left supraclavicular lymph nodes. Serum carcinoembryonic antigen (CEA) was markedly elevated at 126.43 ng/mL, although this finding was not site-specific. Prostate-specific antigen (PSA), alpha-fetoprotein (AFP), CA-125, and parathyroid hormone-related peptide (PTHrP) were non-contributory. Pelvic magnetic resonance imaging (MRI) identified a 1.4 × 1.0 cm PI-RADS 4 lesion in the left posterolateral mid-prostate without extraprostatic extension. Prostate biopsy was deferred given the low PSA level, negative NKX3.1 staining, and overall clinical picture favoring disseminated non-prostatic malignancy. Lumbar spine MRI demonstrated diffuse thoracolumbar metastatic infiltration. The esophageal CT abnormality, considered together with the history of melena, prompted luminal gastrointestinal evaluation. EGD and colonoscopy were then performed. EGD revealed localized ulcerated and erythematous mucosal abnormalities in the middle third of the esophagus, including a small clean-based ulcer, without luminal obstruction or a large mass. Reflux esophagitis and a small hiatal hernia were noted at the gastroesophageal junction (GEJ). Representative endoscopic findings are shown in Figure 2.

**Figure 3:**
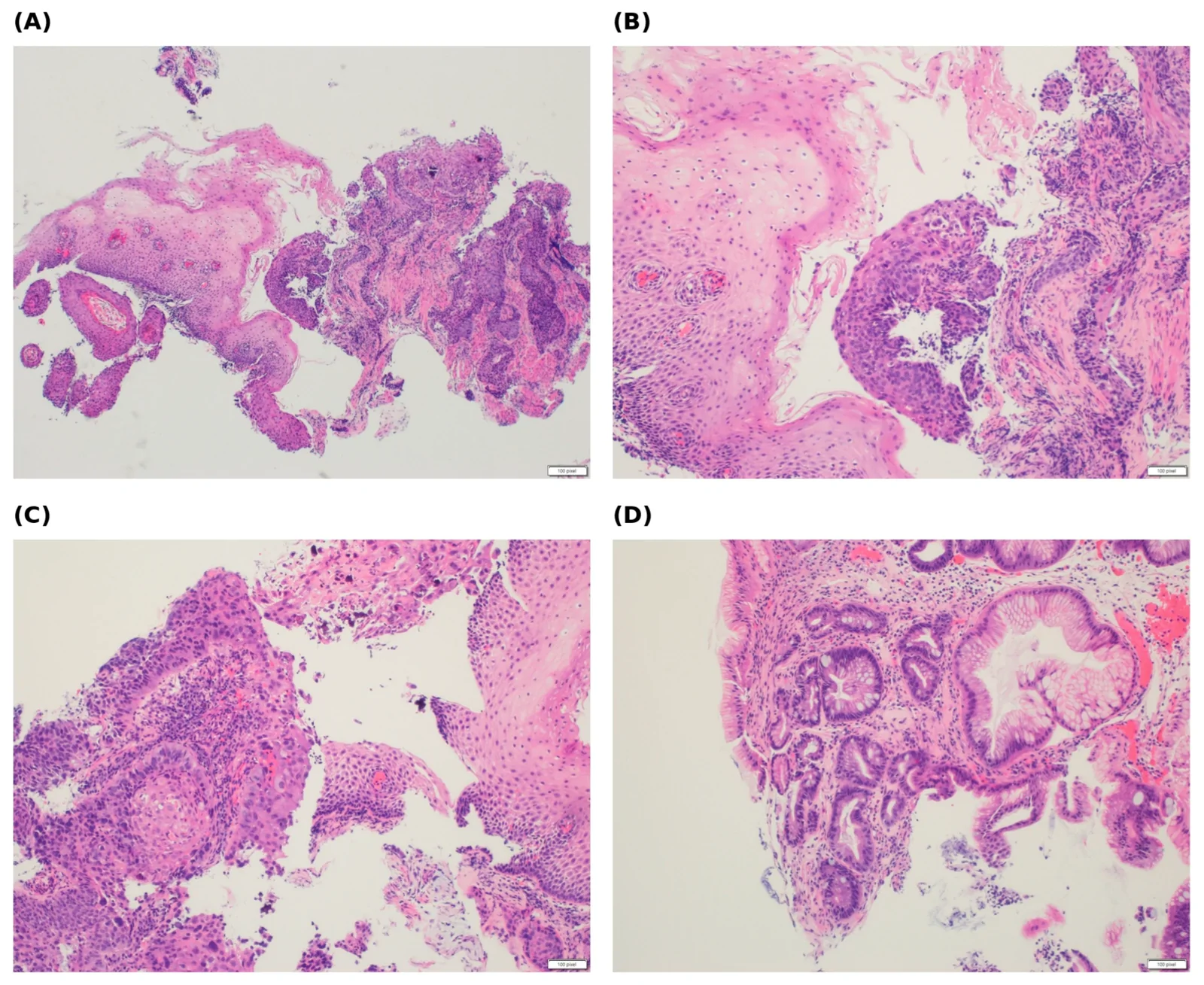
Histopathology of the primary esophageal lesion and GEJ biopsy. (**A**–**C**) H&E sections from the mid-esophageal lesion demonstrating invasive squamous cell carcinoma. (**D**) GEJ biopsy showing gastric-type mucosa with intestinal metaplasia and goblet cells; Barrett’s esophagus criteria were not met in the absence of a documented columnar-lined esophageal segment.

Biopsy of the mid-esophageal lesion confirmed invasive squamous cell carcinoma. Separate GEJ biopsies showed gastric-type mucosa with intestinal metaplasia, goblet cells, and chronic active inflammation. Because no endoscopic evidence of columnar mucosa extending above the GEJ into the tubular esophagus was documented, these biopsies did not fulfill diagnostic criteria for Barrett’s esophagus [14] and were interpreted as incidental findings. Gastric biopsies were without significant pathology; duodenal biopsies showed reactive changes only. Colonoscopy identified tubular adenomas in the ascending and sigmoid colon. Histopathologic findings are shown in Figure 3.

Attribution of the diffuse osseous metastases to the esophageal primary rested on biopsy-proven invasive mid-esophageal squamous cell carcinoma (SCC), a compatible but lineage-indeterminate vertebral metastasis, absence of a more probable primary site on clinical and radiographic evaluation, and a broadly negative IHC profile in the bone biopsy that effectively excluded the most common alternative origins. PD-L1 combined positive score (CPS) was less than 10 [15]. Positron emission tomography–CT (PET-CT) was not performed. Eastern Cooperative Oncology Group (ECOG) performance status was ≥3, precluding systemic chemotherapy or immunotherapy [16,17]. After the ESCC diagnosis was established, palliative external-beam radiation to the lumbar spine (L1 through the sacroiliac joint) was initiated; only 1620 cGy was delivered before treatment was discontinued because of clinical deterioration. The patient was transitioned to hospice and died several weeks after the ESCC diagnosis was established, approximately three months after his initial presentation. The clinical sequence is summarized in Table 1, and the vertebral IHC profile is summarized in Table 2.

## 3. Discussion

This case highlights three interrelated diagnostic challenges: (1) a bone-predominant metastatic syndrome that dominated the early evaluation; (2) a p40-negative vertebral biopsy that did not reveal squamous differentiation; and (3) the need to interpret a negative dysphagia history cautiously in a patient with dementia who nevertheless presented with melena and abdominal symptoms. The central lesson is not that the ESCC was symptom-free, but that symptom reporting was incomplete and that gastrointestinal bleeding and later esophageal wall thickening warranted site-directed endoscopic evaluation.

### 3.1. Clinical Presentation and Diagnostic Sequence

Dysphagia and weight loss dominate the symptomatic presentation of ESCC in large clinical series [1,7,18,19]. In the present case, dysphagia was not documented, but the patient’s dementia limited confidence in a negative symptom history. More importantly, he presented with melena, abdominal pain, nausea, and vomiting. Melena is a gastrointestinal alarm feature and generally warrants evaluation for a bleeding source when clinically appropriate. In retrospect, earlier upper endoscopy could have identified the esophageal lesion sooner. The rationale for not performing EGD during the initial emergency department visit was not available in the record.

The subsequent bone-biopsy-first sequence reflected the order in which the diagnostic clues emerged. At the second presentation, new back pain and CT of the abdomen and pelvis revealed innumerable lytic lesions, while an esophageal abnormality had not yet been identified. Tissue was obtained from the symptomatic L2 lesion during kyphoplasty and radiofrequency ablation. Chest CT later demonstrated esophageal wall thickening with intraluminal debris, after which EGD and direct mucosal biopsy established the primary diagnosis. The radiofrequency ablation performed during the L2 procedure should not be confused with the external-beam palliative radiation administered only after ESCC was confirmed.

### 3.2. Diagnostic Implications of Bone-Predominant ESCC

CUP is formally invoked when histopathology confirms metastatic malignancy but an extensive IHC panel cannot establish a primary site [20]. It accounts for approximately 3–5% of cancer diagnoses and carries a median survival of six to twelve months [21,22]. Contemporary guidelines recommend systematic cross-sectional imaging of the chest, abdomen, and pelvis and site-directed investigation when a suspicious lesion is identified [20,23]. In this case, the CUP designation persisted after the lineage-indeterminate vertebral biopsy until chest CT provided the critical esophageal clue. The case therefore supports prompt endoscopic evaluation when gastrointestinal alarm features or an esophageal imaging abnormality is present, even if the metastatic biopsy is nondiagnostic.

The predominantly osteolytic character of esophageal cancer bone metastases merits emphasis [24]. Clinicians encountering diffuse lytic osseous lesions without a clear primary site will naturally consider multiple myeloma, renal cell carcinoma, and lung or breast carcinoma before an esophageal primary [8,25,26]. In a retrospective series of 58 patients with esophageal cancer and bone metastases, osteolytic lesions were present in 89.7%, skeletal-related events in 91.4%, and median overall survival was five months [9,16,27]. This pattern can closely resemble myeloma or other lytic malignancies. A low threshold for including esophageal and other foregut primaries in the CUP workup is warranted when chest imaging demonstrates a mucosal or mural abnormality in a patient with relevant risk factors [22,23].

### 3.3. p40 Immunohistochemistry and Molecular Adjuncts

p40 is the preferred IHC marker of squamous differentiation, offering superior specificity over p63 across epithelial tumors [10,11]. In ESCC, p40 expression declines with worsening histologic grade: well- and moderately differentiated tumors usually stain strongly, while poorly differentiated tumors may show reduced or absent staining [10]. An analogous grade-dependent loss has been described in other squamous carcinomas [12,28].

A distinct technical mechanism operates in bone specimens. Decalcification is often required for histologic sectioning of mineralized tissue, but hydrochloric acid and prolonged formic acid protocols can degrade antigens and nucleic acids, impairing both IHC and molecular analyses [13]. Ethylenediaminetetraacetic acid-based protocols better preserve antigenicity and DNA quality [13]. Because the decalcification protocol used in this case was not documented, a technical contribution to the p40-negative result cannot be confirmed or excluded. Poor differentiation, decalcification-related loss, or both may explain the absence of p40 staining in a tumor subsequently proven to be squamous cell carcinoma.

The practical implication is that a p40-negative bone biopsy should not terminate the search for a squamous primary when the clinical or imaging context remains suspicious. In that setting, direct sampling of the suspected primary site remains decisive. Tissue-based next-generation sequencing (NGS) of the vertebral specimen could have supplemented IHC by identifying somatic alterations and, if paired with sequencing of the esophageal biopsy, supporting clonal concordance; however, its yield would depend on tumor cellularity, DNA preservation, and the decalcification method [13]. Plasma circulating tumor DNA (ctDNA) NGS could have provided a noninvasive mutation profile, but detection in ESCC is assay- and tumor-burden dependent, and a negative result would not exclude disease [29]. Emerging tissue- and cell-free DNA methylation classifiers can predict tissue of origin in CUP cohorts [30,31], but these assays remain complementary and require further validation for distinguishing specific squamous primary sites. In this case, once CT demonstrated esophageal thickening, EGD with direct mucosal biopsy remained the most efficient and definitive diagnostic test.

### 3.4. Gastroesophageal Junction Intestinal Metaplasia and Barrett’s Esophagus Considerations

GEJ biopsies in this patient showed intestinal metaplasia with goblet cells. Current ACG guidelines require both endoscopic documentation of columnar mucosa extending above the GEJ into the tubular esophagus and histologic confirmation of intestinal metaplasia from that segment to establish a diagnosis of Barrett’s esophagus [22]. No such columnar-lined segment was identified endoscopically; therefore, the biopsy finding alone does not meet diagnostic criteria [14,32]. This distinction matters because attributing the malignancy to a Barrett’s-associated EAC pathway would be histologically inconsistent with confirmed squamous cell carcinoma. The GEJ intestinal metaplasia is most appropriately regarded as incidental in a patient with risk factors for both reflux and tobacco-related mucosal injury.

### 3.5. Limitations

Several limitations warrant acknowledgment. PET-CT was not performed, restricting complete metabolic staging. GEJ biopsies were not accompanied by Prague classification, precluding formal Barrett’s assessment by current endoscopic criteria. Molecular comparison of the vertebral metastasis and esophageal primary was not performed; clonal identity was inferred from clinicopathologic correlation rather than genomic confirmation. The decalcification protocol used for the vertebral specimens was not documented, and information on p40 staining controls, antibody platform, and repeat squamous-marker testing was unavailable. The patient’s dementia limited the reliability of symptom reporting; therefore, the lack of documented dysphagia cannot be interpreted as confirmation that dysphagia was absent. The record also did not document why upper endoscopy was not pursued at the initial presentation despite melena. As a single case report, these observations require validation through larger series or registry-based studies.

Although this patient’s poor performance status precluded systemic therapy, platinum-fluoropyrimidine chemotherapy remains a foundational backbone for chemotherapy-eligible patients with advanced esophageal squamous cell carcinoma and is now commonly combined with immune checkpoint inhibition in the first-line setting, as supported by contemporary guideline recommendations and phase III trial data [1,17,33,34]. This limitation underscores the prognostic importance of timely diagnosis before functional decline forecloses disease-directed therapy.

## 4. Conclusions

ESCC can present as bone-predominant metastatic disease before the primary lesion is recognized. In patients with cognitive impairment, lack of documented dysphagia should not be interpreted as reliable evidence against an esophageal primary. Melena and esophageal mural or mucosal abnormalities should prompt timely EGD, even when metastatic IHC is nondiagnostic. Negative p40 staining in poorly differentiated carcinoma from a bone specimen does not exclude squamous lineage, particularly when decalcification may have compromised antigenicity.

Clinicians evaluating metastatic poorly differentiated carcinoma of uncertain origin should integrate the full clinical sequence, imaging, morphology, IHC, and direct site-specific biopsy. Tissue or liquid-biopsy molecular assays may complement this assessment, but they should not delay endoscopic biopsy of a radiographically suspicious esophageal lesion.

## Figures and Tables

**Table 1:** Patient timeline of key clinical events.

Relative Time	Clinical Event
Initial emergency department presentation	Four to five days of constipation, diffuse abdominal pain, nausea, vomiting, melena, and low-grade fever. Dysphagia/odynophagia was not documented, but the negative history was of uncertain reliability because of dementia. Laboratory evaluation was unrevealing; no imaging or endoscopy was performed; symptoms improved, and the patient was discharged.
Approximately 2 weeks later	Returned with worsening abdominal symptoms and new lumbar/sacral pain. CT abdomen/pelvis showed innumerable lytic osseous lesions and prostatic enlargement. He was transferred to a tertiary center.
Shortly after transfer	The symptomatic L2 lesion underwent kyphoplasty and radiofrequency ablation, with bilateral core biopsies obtained during the same procedural episode. Pathology showed poorly differentiated AE1/AE3-positive carcinoma with negative p40 and other lineage markers, leading to a working diagnosis of CUP.
Subsequent staging and primary-site evaluation	Chest CT showed segmental esophageal wall thickening with intraluminal debris and diffuse osseous metastases. CEA was 126.43 ng/mL. MRI showed diffuse thoracolumbar metastatic infiltration and a small PI-RADS 4 prostate lesion; the overall evaluation did not support a prostatic primary.
After the esophageal CT abnormality was identified	EGD revealed a non-obstructive ulcerated mid-esophageal lesion; colonoscopy showed tubular adenomas.
Pathologic confirmation	Mid-esophageal biopsy confirmed invasive squamous cell carcinoma. GEJ biopsies showed intestinal metaplasia without endoscopic criteria for Barrett’s esophagus.
After ESCC diagnosis	ECOG performance status was ≥3, precluding systemic therapy. Palliative external-beam lumbar radiation was initiated but discontinued after 1620 cGy because of clinical deterioration.
Approximately 3 months after initial presentation	Transitioned to hospice and died several weeks after the ESCC diagnosis.

**Table 2:** Immunohistochemical profile of the L2 vertebral metastasis.

Marker	Result	Interpretation
AE1/AE3 (Pan-cytokeratin)	Positive (diffuse)	Confirms epithelial (carcinoma) lineage
p40 (ΔNp63)	Negative	Squamous marker absent; may reflect poor differentiation and/or decalcification-related antigen loss
CK7	Negative	Argues against CK7-positive adenocarcinoma patterns (lung, pancreatobiliary, breast, gynecologic, upper GI); interpretation limited in poorly differentiated carcinoma
CK20	Negative	Argues against colorectal adenocarcinoma; interpretation limited in poorly differentiated carcinoma
TTF-1	Negative	Argues against lung adenocarcinoma or thyroid carcinoma
GATA-3	Negative	Argues against urothelial or breast carcinoma
PAX8	Negative	Argues against renal, gynecologic, or thyroid carcinoma
NKX3.1	Negative	Argues against prostatic adenocarcinoma
S100	Negative	Argues against melanoma or salivary gland tumors
Overall	Indeterminate/CUP	No lineage-specific differentiation established; esophageal SCC subsequently confirmed by EGD biopsy

## Data Availability

The data supporting the findings of this case report are not publicly available due to patient privacy and ethical restrictions. De-identified data may be available from the corresponding author upon reasonable request and subject to institutional approval.

## Abbreviations

The following abbreviations are used in this manuscript:

**AE1/AE3**	Pan-cytokeratin antibody cocktail
**ACG**	American College of Gastroenterology
**AFP**	Alpha-fetoprotein
**CEA**	Carcinoembryonic antigen
**CPS**	Combined positive score
**CT**	Computed tomography
**CUP**	Carcinoma of unknown primary
**EAC**	Esophageal adenocarcinoma
**ECOG**	Eastern Cooperative Oncology Group
**EDTA**	Ethylenediaminetetraacetic acid
**EGD**	Esophagogastroduodenoscopy
**ESCC**	Esophageal squamous cell carcinoma
**GEJ**	Gastroesophageal junction
**H&E**	Hematoxylin and eosin
**IHC**	Immunohistochemistry
**MRI**	Magnetic resonance imaging
**NCCN**	National Comprehensive Cancer Network
**OS**	Overall Survival
**PET-CT**	Positron emission tomography–computed tomography
**PSA**	Prostate-specific antigen
**PTHrP**	Parathyroid hormone-related peptide
**SCC**	Squamous cell carcinoma
**SEER**	Surveillance, Epidemiology, and End Results
**TTF-1**	Thyroid transcription factor-1
**cfDNA**	Cell-free DNA
**ctDNA**	Circulating tumor DNA
**NGS**	Next-generation sequencing
